# Adversarial robustness improvement for X-ray bone segmentation using synthetic data created from computed tomography scans

**DOI:** 10.1038/s41598-024-73363-2

**Published:** 2024-10-28

**Authors:** Wai Yan Ryana Fok, Andreas Fieselmann, Christian Huemmer, Ramyar Biniazan, Marcel Beister, Bernhard Geiger, Steffen Kappler, Sylvia Saalfeld

**Affiliations:** 1 Faculty of Computer Science, Otto-von-Guericke-University Magdeburg, 39106 Magdeburg, Germany; 2 X-ray Products, Siemens Healthineers AG, 91301 Forchheim, Germany; 3 Institute for Medical Informatics and Statistics, University Hospital Schleswig-Holstein Campus Kiel, 24105 Kiel, Germany

**Keywords:** Robustness, Adversarial Training, Synthetic X-ray, Computed Tomography, Segmentation, Radiography, Biomedical engineering

## Abstract

Deep learning-based image analysis offers great potential in clinical practice. However, it faces mainly two challenges: scarcity of large-scale annotated clinical data for training and susceptibility to adversarial data in inference. As an example, an artificial intelligence (AI) system could check patient positioning, by segmenting and evaluating relative positions of anatomical structures in medical images. Nevertheless, data to train such AI system might be highly imbalanced with mostly well-positioned images being available. Thus, we propose the use of synthetic X-ray images and annotation masks forward projected from 3D photon-counting CT volumes to create realistic non-optimally positioned X-ray images for training. An open-source model (TotalSegmentator) was used to annotate the clavicles in 3D CT volumes. We evaluated model robustness with respect to the internal (simulated) patient rotation $\alpha$ on real-data-trained models and real&synthetic-data-trained models. Our results showed that real&synthetic- data-trained models have Dice score percentage improvements of 3% to 15% across different $\alpha$ groups compared to the real-data-trained model. Therefore, we demonstrated that synthetic data could be supplementary used to train and enrich heavily underrepresented conditions to increase model robustness.

## Introduction

In clinical imaging examinations, proper patient positioning is crucial for accurately capturing anatomical structures. This can aid in medical image analysis^1^, longitudinal disease monitoring, radiotherapy planning^2^, thereby enhancing diagnostic confidence. To ensure correct patient positioning, an automatic positioning analysis system could be developed to support clinicians and technical assistants, so to optimize patient management by reducing the time or need for retakes. There are several quality criteria for patient positioning in chest X-rays (CXRs), such as the clavicle-spine distance and the visibility of certain thoracic anatomical landmarks^3^. A segmentation approach could identify the key anatomical structures in the images, allowing for the computation of quality metrics like distances and overlaps between anatomies.

Deep learning has emerged as a powerful tool for medical image analysis, ranging from segmentation, disease detection to report writing. It relies on large quantities of annotated images and could be the basis for the aforementioned segmentation task. However, obtaining large-scale annotated clinical CXRs, especially those with non-optimal positioning, is challenging. In addition, the robustness of current segmentation models in adversarial-positioned CXR is unknown. In realm of this chicken-and-egg dilemma, we first explore the robustness current segmentation models, and then supplementary train the models with adversarial CXR to improve robustness.

In order to reduce vulnerability to adversarial attacks when deploying deep learning models in real-world applications, robustness certification and adversarial training have been studied over the years to enhance model resilience. Robustness is the degree that a model’s performance changes in the presence of perturbations or uncertainties. Some studies certified robustness by using an abstract domain such as Zonotope to capture the effect of affine transformations inside neural networks^4,5^. On the other hand, various methods have been suggested to generate adversarial perturbations with respect to the input and learned features^6–9^, including fast gradient sign method^10^, DeepFool^11^, saliency map attacks^12^, expectation over transformation^13^ and curriculum adversarial training^14^. Studies have been conducted that apply the adversarial pertubations in natural red-blue-green images^15^ and skin lesion classification in medical imaging^16,17^. Besides classification, robustness benchmarking was also demonstrated by crafting adversarial examples using fast gradient sign method, DeepFool and saliency map attacks on whole brain segmentation^18^.

There is an emerging usage of realistic synthetic data for machine learning in medicine^19–22^, as curation of large-scale annotated clinical data is challenging due to scarcity or ethical issues, especially adversarial data. Synthetic image generation was studied in a range of imaging modalities including pathological images on skin lesions^23,24^, retinal images^25^, and in generation of synthetic CT images from MR images^26–29^. Particularly in X-ray imaging, synthetic X-rays (also known as digitally reconstructed radiographs, DRR)^30^ can be also generated from 3D CT volumes by analytic forward projection or GANs. Only a few studies have been carried out for using synthetic X-rays as training, for example to detect lung lesions^31^ or to quantify patient rotation^32^. A CNN trained with synthetic X-rays from CT volumes to quantify airspaces achieved an accuracy on the level of radiologists for a COVID lesion segmentation task^33^. Gao et al.^34^ used synthetic X-rays for lesion segmentation, landmark detection and surgical tool detection tasks, and their ground truth annotations were obtained by automatic segmentation or forward kinematics.

We propose to generate synthetic X-ray images from 3D CT volumes also for the use case to generate large amount of normal and adversarial x-ray images and in-image ground truth annotations systematically at the same time. We used a state-of-the-art CT segmentation tool TotalSegmentator^35^ to obtain ground truth for left and right clavicles in 3D CT domain. Both 3D image and annotations were forward projected to 2D X-ray domain and are characterized by non-optimal patient positioning. We trained clavicle segmentation models using real data and additionally with synthetic data for robustness evaluation. A model is rated robust if the Dice scores are consistent to slight changes in adversarial features, and in this study, we evaluated the Dice scores across different projection angles. We further evaluated the performance of open available CXR segmentation model TorchXRayVision^36^ as baseline comparison.

Our contribution in this paper is threefold: We first demonstrated the generation of synthetic CXR and their corresponding segmentations from CT volumes. Subsequently, we explored two distinct applications of synthetic images: as a mean to test and interpret model performance on adversarial data, and to augment an existing training dataset with synthetic adversarial cases, thereby enhancing model performance and robustness. Moreover, we used the open source models TotalSegmentator and TorchXRayVision to enable a more reproducible research.

## Methods

### Overview

**Fig. 1:**
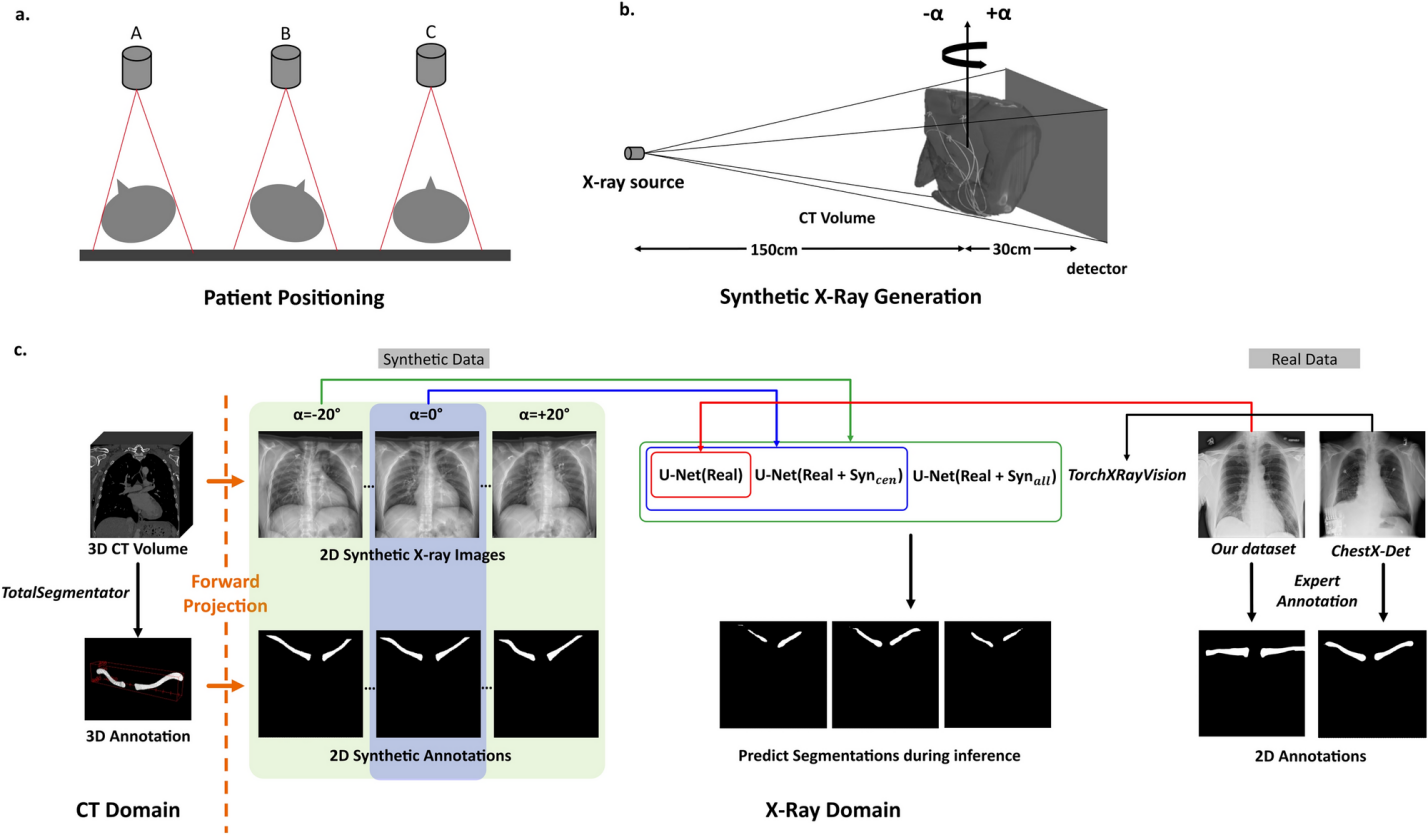
(**a**) Patient positioning possibilities in clinical X-Ray examination, A and B represent adversarial positioning and C represents correct positioning; (**b**) Simulation setup for generating rotated and non-rotated synthetic X-rays with an angle $\alpha$ in the range of [$-20^{\circ }$, $20^{\circ }$]; (**c**) Our proposed workflow: In the CT domain, we generated ground truth segmentation masks using TotalSegmentator. Paired CT image and segmentation volumes were forward projected to X-ray domain as normal and adversarial data for training and testing, and to quantify robustness in segmentation models TorchXRayVision, U-Net(Real), U-Net($\hbox {Real} + \hbox {Syn}_{{cen}}$) and U-Net($\hbox {Real} + \hbox {Syn}_{{all}}$).

Figure 1 shows our concept and X-ray simulation. It shows the forward projection setup for generating synthetic X-ray images (Figure 1a). Figure 1b illustrates how rotated (adversarial) and non-rotated (normal) data are position by rotating patient volume along y-axis. Figure 1c shows the pipeline from synthetic X-ray images and annotations to adversarial robustness measurement.

### Generation of synthetic X-ray images and ground truth masks from computed tomography scans

A total of 116 photon-counting CT (NAEOTOM Alpha, Siemens Healthineers AG, Forchheim, Germany) datasets from individual patients were used to generate synthetic X-ray images. Each CT volume has a voxel size of 0.5$\times$0.5$\times$0.7$\hbox {mm}^3$, and $\approx$1000 slices. Each CT volume underwent forward projection by ray tracing based on a cone-beam geometry. Virtual X-ray beams traverse the CT volume, and interacts with internal anatomies as attenuation and scattering. By accumulating the attenuation values along these paths, a simulated X-ray image is generated, representing the intensity of X-ray transmission through the volume from various angles^37^. With parameters similar to clinical chest X-ray examinations^38^, the X-ray source-to-patient distance is 150 cm, patient-to-detector distance is 30 cm, and the simulated detector has a matrix of $1800 \times 1800$ pixels. To simulate the adversarial patient positioning, projection parameters were set such that X-ray source is rotated along the y-axis with angle $\alpha$ in range of [$-20^{\circ }$, $20^{\circ }$], with a step size of $2^{\circ }$ and the central projection at $0^{\circ }$ (Figure 1b). Furthermore, standard radiographic image post-processing was applied to images.

To generate the synthetic ground truth annotations, each photon-counting CT dataset was segmented by the open-source deep learning segmentation toolbox TotalSegmentator^35^ (version 2.0.5). TotalSegmentator could segment 104 anatomic structures in CT images and was trained on nnUNet^39^ using 1204 patient datasets. Python-API was used to call TotalSegmentator and ’roi-subset’ option was used to segment left and right clavicles separately. The resulting left and right volumes were combined such that each photon-counting CT has a corresponding 3D segmentation volume of clavicles (Fig. 1c). The annotations were then forward projected using the same setup and parameters as in their image domain, and binarized to obtain the segmentation mask.

### Datasets and training

TorchXRayVision^36^ is a Python library that contains chest X-ray datasets and models including disease classification and segmentation. The segmentation model in TorchXRayVision was trained by the ChestX-Det^40^, which is a subset of the NIH ChestX-14 dataset. ChestX-Det contains 3575 images with segmentation annotations for chest anatomies including clavicles and lung, which were annotated by three board certified radiologists. The chest segmentation model in TorchXRayVision used a pretrained PSPNet^41^ and was trained with 3575 images. Instead of only using only an internal model, we included TorchXRayVision model as a baseline and to enable reproducible research.

**Table 1:** Dataset distribution for public and self-trained models.

	Training			Testing	
	Real	Synthetic	Total	Real	Synthetic
TorchXRayVision	3575	0	3575	350	420
U-Net(Real)	3434	0	3434		
U-Net(Real+Syn_cen)	3434	288	3731		
U-Net(Real+Syn_all)	3434	2016	5450		

For our own segmentation model, the real X-ray dataset consists of 3434 CXR images, with clavicle masks annotated manually by experts. 350 real CXR images were randomly separated for testing. A total of 3434 images were used for training (train: 3134, valid: 387). From the 116 photon-counting CT examinations, we generated 21 synthetic X-ray images for each dataset, yielding a total of 2436 synthetic X-ray images and the corresponding clavicle annotations. Subject-specific splitting were performed to randomly select 96 datasets (2016 images) for training and 20 datasets (420 images) for testing. With an addition of 288 well-positioned synthetic X-ray images, U-Net($\hbox {Real} + \hbox {Syn}_{{cen}}$) has a total of 3731 training images. U-Net($\hbox {Real} + \hbox {Syn}_{{all}}$) uses 2016 synthetic well- and adversarial- positioned X-ray images, resulting in a total of 5450 training images. Table 1 provides detailed information about the dataset and the models. Same image preprocessing steps were applied to real and synthetic X-ray images before training, which include resize and normalization. Both real and synthetic X-ray and respective clavicle masks were resized to an image dimension of $256 \times 256$ by bilinear interpolation and zero padding. Subsequently, the pixel values in real and synthetic images were normalized into the range of [0, 1]. U-Net^42^ was used to train our models, Dice loss and Adam optimizer with a learning rate of 0.01 were used and early stopping was applied when the model did not improve in the last 30 epochs.

### Robustness evaluation

An ablation study was used to select the optimal network hyperparameters. By varying the batch sizes, depth of U-Net and loss functions, the Dice scores were evaluated. For the loss function in the network, $1-$ Dice similarity coefficient^43^ resulted as Dice loss^44^ calculation:

1 $$\begin{aligned} DiceLoss (y,{\hat{y}}) = 1 - \frac{2y{\hat{y}}+\varepsilon }{y+{\hat{y}}+\varepsilon }, \end{aligned}$$

where *y* indicates the ground truth, ${\hat{y}}$ indicates the predicted segmentation, and $\varepsilon$ is used to avoid division by 0 so to ensure loss function stability. Multiplication of *y* and ${\hat{y}}$ indicate the intersected region of ground truth and predicted segmentation. Whereas the Dice with binary cross entropy (BCE) loss is defined as:

2 $$\begin{aligned} BCELoss (y,{\hat{y}})= & -ylog({\hat{y}}) + (1-y)log(1-{\hat{y}}) , \end{aligned}$$

3 $$\begin{aligned} DiceBCE= & BCELoss + DiceLoss , \end{aligned}$$

both Dice and DiceBCE loss functions were evaluated in the ablation study. The real and synthetic X-ray images were evaluated on TorchXRayVision, images were resized to $512 \times 512$ and normalized to [-1024, 1024] as in their pipeline. The first two classes in the output predictions among 14 classes are left and right clavicles respectively, and combined to form the clavicle prediction. Both real and synthetic X-ray images were evaluated on our self-trained models U-Net(Real), U-Net($\hbox {Real} + \hbox {Syn}_{{cen}}$) and U-Net($\hbox {Real} + \hbox {Syn}_{{all}}$). For each model’s output, the prediction was defined as the true class when the predicted probability is >0.5.

Robustness was shown as Dice score in a boxplot for 20 randomly selected patients across 21 projections, resulting 420 images. For simpler representation, internal patient rotation ${\alpha }$ values are divided into five groups. Well-positioned X-ray images denoted as group “Center” ($-2^{\circ }$ to $2^{\circ }$). Adversarial images are categorized into “Moderate Negative” ($-20^{\circ }$ to $-12^{\circ }$), “Low Negative” ($-10^{\circ }$ to $-4^{\circ }$), “Low Positive” ($4^{\circ }$ to $10^{\circ }$), and “Moderate Positive” ($12^{\circ }$ to $20^{\circ }$). Interquartile range (IQR) is represented by the upper and lower box edges which indicates 75th percentile (or third quartile, Q3) and 25th percentile (or first quartile, Q1) respectively. The whiskers extend to the farthest data point lying within 1.5 $\times$ IQR from the box, with upper whiskers $= Q3 + (1.5 \times IQR)$, while lower whiskers $= Q1 - (1.5 \times IQR)$. Statistical analysis on the mean Dice score and standard deviation for four models were evaluated on synthetic test images, and real test images as a baseline. To further demonstrate the Dice changes for patients at different angles, we performed a box plot comparing U-Net(Real) with U-Net($\hbox {Real}+\hbox {Syn}_{{cen}}$) or U-Net($\hbox {Real}+\hbox {Syn}_{{all}}$) on angles $-20^{\circ }$, $-10^{\circ }$, $0^{\circ }$, $10^{\circ }$ and $20^{\circ }$.

Furthermore, the distance metrics Hausdorff Distance (HD) and the 95th percentile of Hausdorff Distance (HD95) are also evaluated. The ground truth *Y* and predicted segmentation ${\hat{Y}}$ could be represented by respective point sets $Y = \{y_1, y_2, . . . , y_n\}$ and ${\hat{Y}} = \{{\hat{y}}_1, {\hat{y}}_2, . . . , {\hat{y}}_m\}$. It is a measure of the distance between two subsets of a metric space. With $\parallel y-{\hat{y}} \parallel _2$ is the Euclidean distance between *y* and ${\hat{y}}$, the Hausdorff Distance $D(Y, {\hat{Y}})$ of *Y* to ${\hat{Y}}$ is defined as:

4 $$\begin{aligned} \begin{aligned} d(Y, {\hat{Y}})&= \max _{y \in Y} \min _{{\hat{y}} \in {\hat{Y}}} \parallel y-{\hat{y}} \parallel _2 \\ HD(Y, {\hat{Y}})&= \max (d(Y, {\hat{Y}}), d({\hat{Y}}, Y)) \end{aligned} \end{aligned}$$

Instead of the maximum of the nearest point between *Y* and ${\hat{Y}}$, HD95 is defined as 95th percentile of the distances. This can reduce the sensitivity to outliers and provide a more robust comparison.

## Results

### Performance of real data-trained models

**Fig. 2:**
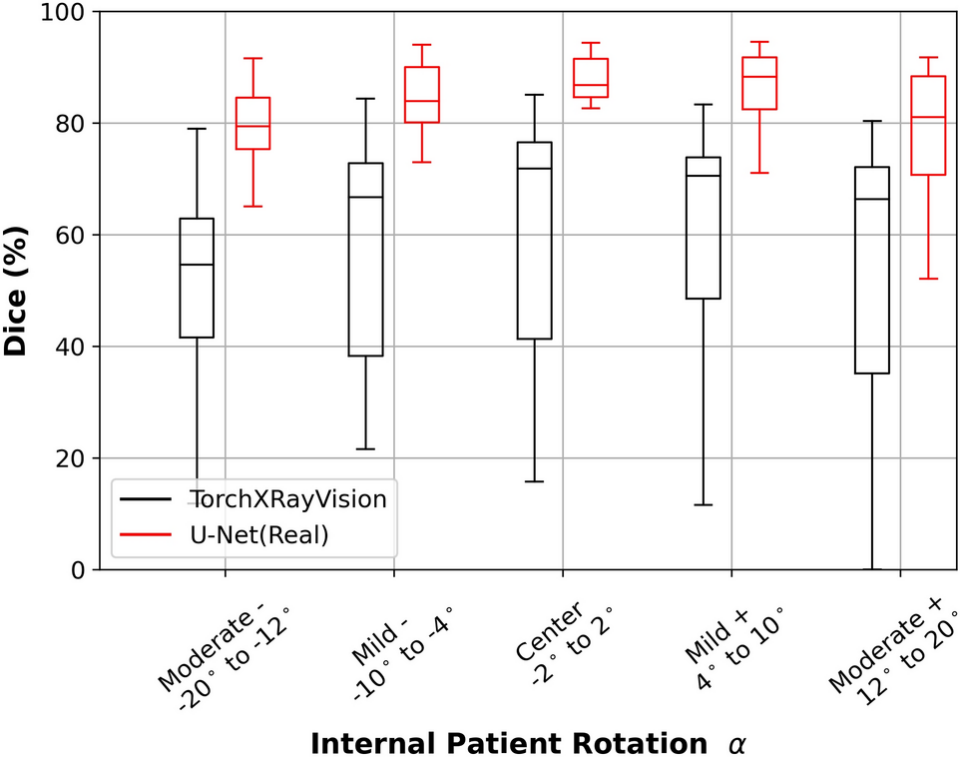
Robustness as Dice score for real data-trained segmentation models in boxplot on well- and adversarial- positioned data. TorchXRayVision is black and U-Net(Real) is red in color. Internal patient rotation ${\alpha }$ values are divided into five groups. Well-positioned X-ray images are grouped as center. While adversarial data indicated by Moderate Negative (-), Low Negative (-), Low Positive (+), and Moderate Positive (+). The ranges includes ${\alpha }$ values with a step size of $2^{\circ }$. Ie. center represent ${\alpha }$ values of $-2^{\circ }$, $0^{\circ }$, and $2^{\circ }$.

Figure 2 shows the robustness analysis of models trained with real X-ray images as a boxplot, robustness for TorchXRayVision is black and U-Net(Real) is red in color. Internal patient rotation ${\alpha }$ values are divided into five groups. Well-positioned X-ray images are grouped as center. The boxplot shows a clear, symmetrically distributed trend across the five groups of ${\alpha }$ values, delineated by a curve-shaped progression in terms of central tendency measures and quartile ranges. Highest median values are observed in central ${\alpha }$ values for both TorchXRayVision and U-Net(Real) model, as depicted by the line within the box. Mild negative and positive ${\alpha }$ demonstrate successively lower median, while moderate negative and positive ${\alpha }$ exhibit the lowest median values. A similar pattern is also observed in Table 2 which shows the Dice score, HD and HD95 in mean and standard deviation. Interquartile range (IQR) is represented by the upper and lower box edges which indicates 75th and 25th percentile respectively. IQR are large in moderate negative and positive ${\alpha }$ values, while narrowing towards the central ${\alpha }$ values. This pattern suggests a decrease in variability towards X-rays positioned closer to the center. This pattern is further supported by the whiskers, which extend to the farthest data point lying within 1.5 $\times$ IQR from the box. The upper and lower whiskers for both models exhibit a consistent decrease from the central towards the outer ${\alpha }$ values. Notably, the moderate negative and positive ${\alpha }$ values exhibit large distance in their lower whiskers. This highlighted the increase in model performance variability at higher ${\alpha }$ values. Overall, the observed curve-shaped pattern in median and quartile range, coupled with the increasing distances of the whiskers, underscores the decrease in robustness of models in adversarial data.

**Table 2:** Robustness of four models. Dice score, Hausdorff Distance (HD) and 95th percentile of HD (HD95) are measured between the ground truth and predicted segmentation by four models on synthetic data.

		Moderate -	Low -	No	Low +	Moderate +
TorchXRayVision	Dice	48.67 ± 21.86	58.06 ± 21.53	62.26 ± 20.32	60.21 ± 22.25	53.27 ± 26.12
	HD	2077.14 ± 115.53	2219.49 ± 32.33	2287.61 ± 32.06	2285.14 ± 41.69	2176.00 ± 82.62
	HD95	1539.31 ± 100.88	1796.58 ± 90.51	1913.68 ± 49.05	1870.52 ± 84.12	1630.44 ± 45.73
U-Net(Real)	Dice	79.12 ± 8.90	82.94 ± 10.46	83.79 ± 13.24	86.37 ± 6.61	76.71 ± 17.69
	HD	6.91 ± 0.30	6.38 ± 0.11	6.31 ± 0.12	6.14 ± 0.17	6.69 ± 0.35
	HD95	5.07 ± 0.23	4.38 ± 0.10	4.15 ± 0.19	4.01 ± 0.03	4.84 ± 0.39
U-Net($\hbox {Real}+\hbox {Syn}_{{cen}}$)	Dice	87.14 ± 4.58 (10.14%)	88.84 ± 4.52 (7.11%)	**89.61 **± **2.78** **(6.95%)**	89.14 ± 3.30 (3.21%)	86.02 ± 7.57 (12.14%)
	HD	5.57 ± 0.27	5.43 ± 0.10	5.56 ± 0.08	5.64 ± 0.10	5.76 ± 0.13
	HD95	4.08 ± 0.24	3.77 ± 0.03	**3.63 **± **0.10**	3.78 ± 0.06	4.07 ± 0.39
U-Net($\hbox {Real}+\hbox {Syn}_{{all}}$)	Dice	**88.50** ± **3.45** **(11.86%)**	**89.03 **± **3.47** **(7.34%)**	89.21 ± 3.49 (6.47%)	**89.32 **± **2.19** **(3.42%)**	**88.95 **± **3.39** **(15.96%)**
	HD	**5.36 **± **0.10**	**5.37 **± **0.09**	**5.36 **± **0.07**	**5.50 **± **0.06**	**5.40 **±**0.04**
	HD95	**3.71 **± **0.10**	**3.67 **± **0.04**	**3.63 **± **0.05**	**3.64 **± **0.06**	**3.62 **± **0.07**

Values are shown in groups of normal and adversarial data using the internal rotation feature ${\alpha }$. Values are shown as mean ± standard deviation. Values in bracket show the percentage increase of the real and synthetic data-trained models compared to real data-trained model U-Net(Real). Bold values indicate the best results for each internal rotation category.

**Fig. 3:**
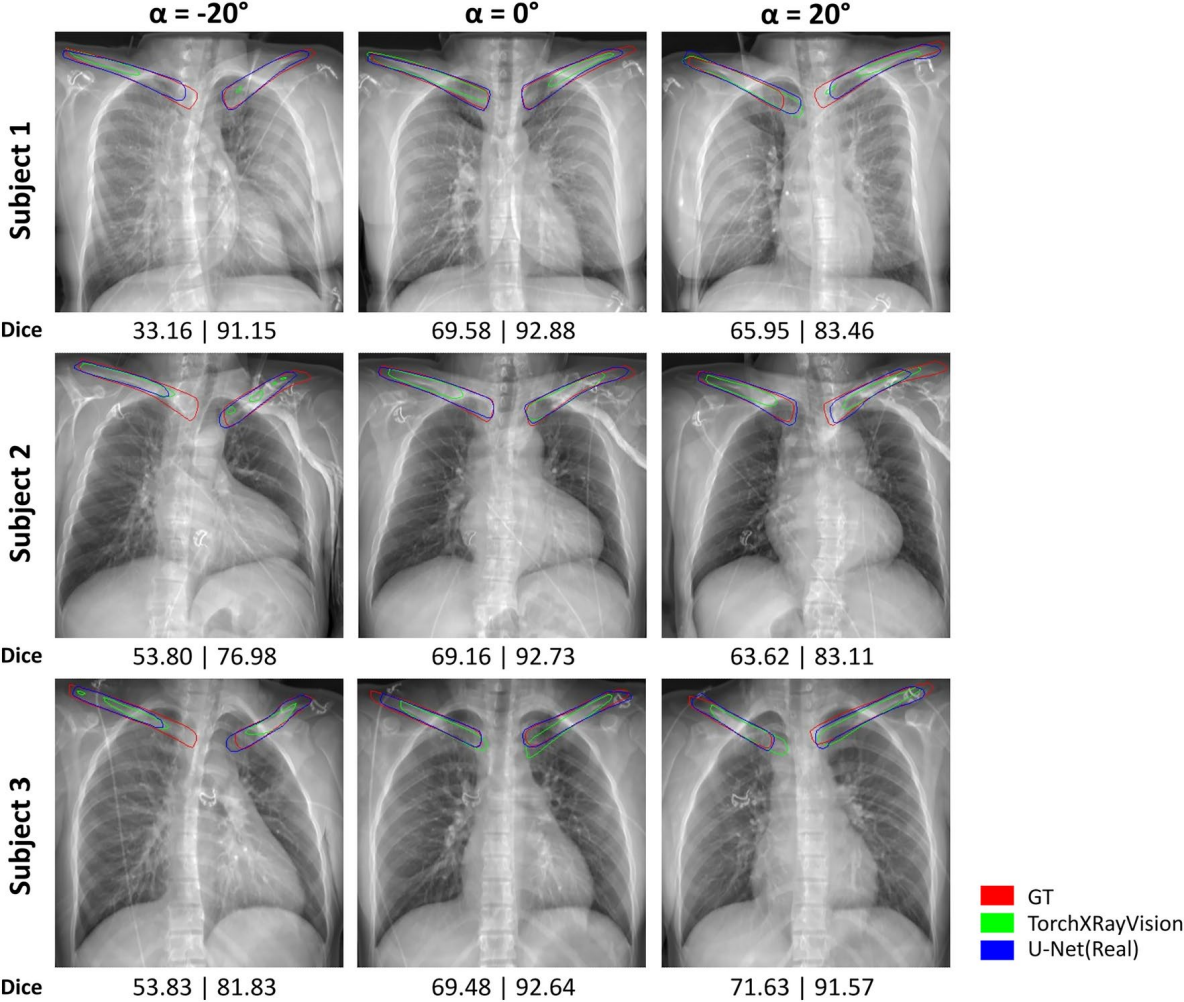
Clavicle segmentation results of real-data-trained segmentation models. Three subjects with adversarial-positioned ($\alpha =-2^{\circ }$ and $20^{\circ }$) and well-positioned ($\alpha =0^{\circ }$) images are shown.The ground truth (GT) segmentation contours from TotalSegmentator are red, TorchXRayVision are green, and U-Net(Real) are blue in color. The Dice score analysis with respect to GT for each patient and angle are shown below each image, left is TorchXRayVision and right is U-Net(Real).

Figure 3 shows synthetic X-ray of three test subjects with segmentation contours of ground truth (red color) and predictions from TorchXRayVision (green color) and U-Net(Real) (blue color). In ${\alpha } = 0^{\circ }$, both TorchXrayVision and U-Net(Real) segmentation contour are most similar to the ground truth masks. In most cases of ${\alpha } = -20^{\circ }$ and $20^{\circ }$, the clavicle contours for both models are either longer or shorter than the ground truth.

### Improvement when trained with real and synthetic adversarial data

**Fig. 4:**
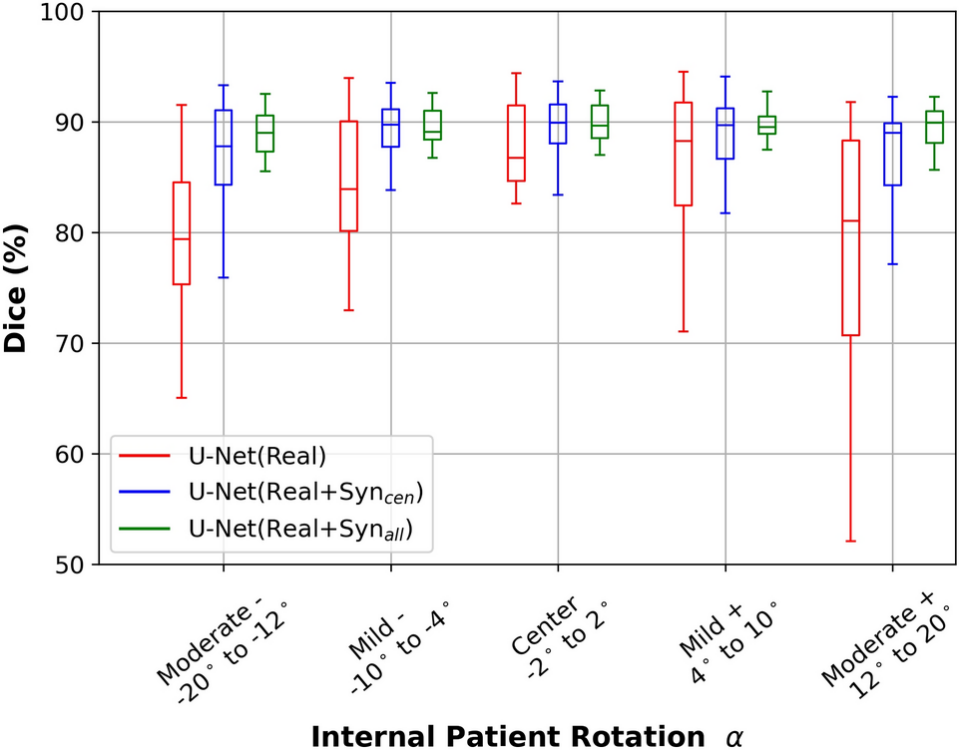
Robustness as Dice scores for real and synthetic data-trained segmentation models in boxplot on well- and adversarial- positioned data. Values in mean and standard deviation are shown in Table 2. U-Net(Real) is red, U-Net($\hbox {Real}+\hbox {Syn}_{{cen}}$) is blue and U-Net($\hbox {Real}+\hbox {Syn}_{{all}}$ is green in color. Description for the boxplot is similar as Fig. 2.

To assess the influence of the synthetic data, we further trained the U-Net model with real and synthetic X-ray images, as U-Net($\hbox {Real}+\hbox {Syn}_{{cen}}$) and U-Net($\hbox {Real}+\hbox {Syn}_{{all}}$). Figure 4 shows the robustness analysis of models trained with real X-ray images as a boxplot, robustness for U-Net($\hbox {Real}+\hbox {Syn}_{{cen}}$) is blue and U-Net($\hbox {Real}+\hbox {Syn}_{{all}}$) is green in color. There is a consistent increase in the median in both U-Net($\hbox {Real}+\hbox {Syn}_{{cen}}$) and U-Net($\hbox {Real}+\hbox {Syn}_{{all}}$) models compared to U-Net(Real). The IQR and the distance between the upper and lower whiskers exhibit a consistent decrease across all five groups of ${\alpha }$ values when synthetic data is incorporated into training for both U-Net($\hbox {Real}+\hbox {Syn}_{{cen}}$) and U-Net($\hbox {Real}+\hbox {Syn}_{{all}}$). And the IQR and whiskers range in U-Net($\hbox {Real}+\hbox {Syn}_{{all}}$) even reduced more than U-Net($\hbox {Real}+\hbox {Syn}_{{cen}}$). This indicates a positive impact of adversarial synthetic data incorporation on model performance.

Furthermore, when considering the trend across various ${\alpha }$ values, the median, IQR and whiskers ranges of U-Net($\hbox {Real}+\hbox {Syn}_{{all}}$) are equally consistent across all ${\alpha }$ groups. While the symmetric curve trend still exists in both U-Net($\hbox {Real}+\hbox {Syn}_{{cen}}$) and U-Net(Real). The two models trained with only well-positioned data have lower median in moderate negative and positive than center ${\alpha }$ value. Also, the IQR has a larger decrease in moderate ${\alpha }$ than in mild ${\alpha }$ values, with most pronounced decrease in mild positive. While in center ${\alpha }$ values, the IQR of U-Net($\hbox {Real}+\hbox {Syn}_{{cen}}$) and U-Net($\hbox {Real}+\hbox {Syn}_{{all}}$) are similar. The above results collectively underscore the efficacy of synthetic adversarial data-incorporated training, which enhanced the resilience and robustness of deep learning segmentation models. In addition, similar pattern are observed quantitatively in Table 2. Percentage increase in Dice score are larger in moderate than center or low ${\alpha }$ values.

**Fig. 5:**
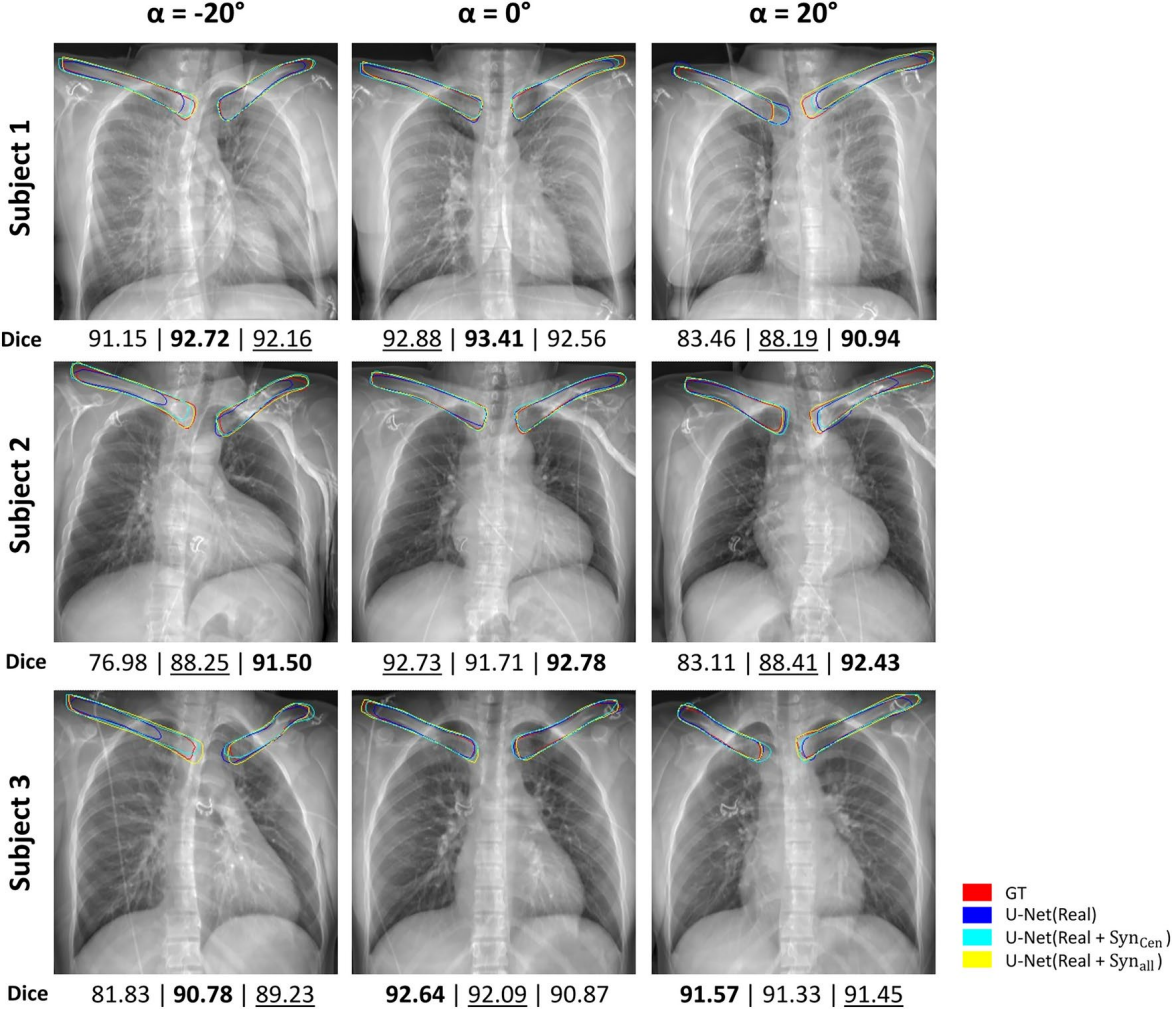
Clavicle segmentation results of real and synthetic data-trained segmentation models. Three subject images where the patient is adversarial-positioned ($\alpha =-20^{\circ }$ and $20^{\circ }$) and well-positioned ($\alpha =0^{\circ }$). The segmentation contours color for ground truth (GT) are red, U-Net(Real) are blue and U-Net($\hbox {Real}+\hbox {Syn}_{{cen}}$) are cyan, and U-Net($\hbox {Real}+\hbox {Syn}_{{all}}$) are yellow. The Dice score analysis with respect to GT for each patient and angle are shown below each image, left is U-Net(Real), middle is U-Net($\hbox {Real}+\hbox {Syn}_{{cen}}$), and right is U-Net($\hbox {Real}+\hbox {Syn}_{{all}}$). Bold indicates the highest value among the triplets, and underline indicates the second-highest value.

Figure 5 shows synthetic X-ray of three test subjects with segmentation contours of ground truth in red, predictions from U-Net(Real) is blue, U-Net($\hbox {Real}+\hbox {Syn}_{{cen}}$) is cyan, and U-Net($\hbox {Real}+\hbox {Syn}_{{all}}$) is yellow in color. In ${\alpha } = 0^{\circ }$, the segmentation contours from all three models have a high similarity to the ground truth, with U-Net(Real) and U-Net($\hbox {Real}+\hbox {Syn}_{{cen}}$) slightly under or over predict on Subject 1, and 3. In ${\alpha } = -20^{\circ }$ and $20^{\circ }$, the contour U-Net(Real) fall short in at least one clavicle in each subject. Moreover, U-Net($\hbox {Real}+\hbox {Syn}_{{all}}$) has generally a higher similarity to ground truth than U-Net($\hbox {Real}+\hbox {Syn}_{{cen}}$). This is supported by the Dice score analysis shown below each image. U-Net($\hbox {Real}+\hbox {Syn}_{{all}}$) generally shows the highest or second-highest Dice score.

### Network and ablation study

The training of the three models was performed on a NVIDIA A40 48GB GPU. Each epoch takes $\approx$300 seconds, early stopping was applied when model did not improve within the last 30 epochs. An ablation study was performed on the U-Net(Real) where different components or loss terms are tested during training to evaluate the efficacy of each component. By varying the batch sizes, depth of U-Net and loss functions, the Dice score were evaluated. Table 3 shows the Dice score in mean and standard deviation of the ablation study, bold values indicates the highest Dice. The hyperparameters with highest Dice score is when using Dice loss, batch size of 32 and 5 layer in U-Net. Hence, these hyperparameters were also used in U-Net($\hbox {Real}+\hbox {Syn}_{{cen}}$) and U-Net($\hbox {Real}+\hbox {Syn}_{{all}}$) training. As a baseline comparison, we also test the other three models TorchXRayVision, U-Net($\hbox {Real}+\hbox {Syn}_{{cen}}$) and U-Net($\hbox {Real}+\hbox {Syn}_{{all}}$) on 350 real X-ray images. Respective Dice scores for four models are shown in Table 4.

**Table 3:** Ablation study on U-Net(Real). 18 combinations resulted when varying depth of U-Net, loss function and batch size. Dice score were shown as mean and standard deviation (SD). Bold indicates highest Dice score.

Exp	1	2	3	4	5	6	7	8	9
Depth	3						4		
Loss	Dice			DiceBCE			Dice		
Batch	8	16	32	8	16	32	8	16	32
Mean	89.79	89.88	90.00	89.42	89.28	88.60	91.63	91.37	91.33
SD	10.84	11.08	9.24	10.26	11.45	9.78	8.68	8.44	8.82

Exp	10	11	12	13	14	15	16	17	18
Depth	4			5					
Loss	DiceBCE			Dice			DiceBCE		
Batch	8	16	32	8	16	32	8	16	32
Mean	91.17	91.45	91.23	92.04	92.14	**92.58**	92.26	91.53	90.96
SD	8.12	8.48	9.00	7.07	7.05	**7.14**	7.55	7.58	7.38

**Table 4:** Evaluation of four models on real X-ray images. Dice score were shown as mean and standard deviation (SD). Bold indicates highest Dice score.

	TorchXRayVision	U-Net(Real)	U-Net($\hbox {Real} + \hbox {Syn}_{{cen}}$)	U-Net($\hbox {Real} + \hbox {Syn}_{{all}}$)
Mean	42.03	**92.58**	91.96	92.30
SD	32.62	**7.14**	9.47	8.29

## Discussion

To separate the effect of domain learning when measuring adversarial robustness, we define the model U-Net($\hbox {Real} + \hbox {Syn}_{{cen}}$) for which only well-positioned synthetic X-ray were added to training. This model did not seen any adversarial data. The increase in median, and reduction of IQR and whiskers range in U-Net($\hbox {Real} + \hbox {Syn}_{{cen}}$) than U-Net(Real) across all ${\alpha }$ values demonstrated the transfer learning between real and synthetic X-ray. However, U-Net($\hbox {Real} + \hbox {Syn}_{{cen}}$) still exhibit a curve shape trend, ie. higher Dice in the center while lower Dice on the large ${\alpha }$ values. In addition, the curve shape trend still exists when using the open-source TorchXRayVision as a baseline comparison. Yet, the primary focus of this analysis is not on the overall and absolute performance of different network, but rather on observing the trend of the Dice score across various adversarial data, ie. angles. We can see there is an agreement of the Dice score trend across different angles which agrees to our model trained with internal data.

Compared to U-Net($\hbox {Real} + \hbox {Syn}_{{cen}}$), U-Net($\hbox {Real} + \hbox {Syn}_{{all}}$) has a reduced whisker range in all groups and reduction in IQR in moderate negative, mild and moderate positive angles. With only 96 images added per adversarial features, angle ${\alpha }$, to the training, the spread of Dice score reduced and the model performances improved. Furthermore, U-Net($\hbox {Real} + \hbox {Syn}_{{all}}$) has similar median Dice, IQR and whisker range for all ${\alpha }$ angles. Robustness refers to the ability of a model to maintain its performance and generalization capabilities when faced with perturbations. Hence, similar variability shows the resilience and reduced misclassification in clavicle segmentations. On the other hand in the center $\alpha$ group, both U-Net($\hbox {Real} + \hbox {Syn}_{{cen}}$) and U-Net($\hbox {Real} + \hbox {Syn}_{{all}}$) have similar IQR and median, but slightly more reduced whiskers range. Both models contain the same well-positioned synthetic X-ray images, while U-Net($\hbox {Real} + \hbox {Syn}_{{all}}$) contains additional adversarial-positioned synthetic images. With addition of adversarial training data, the peripheral Dice score is reduced, thus indicating the learning of adversarial features. As a rule of thumb, the increase in training data might contribute to the combined effect of the performance improvement. Yet the performance improvement in the center category is not substantial, for instance in Figure 4, the blue box and green box in the center category even with an increase of total training data from 3731 to 5450. The increase of performance only appears in adversarial angles. This suggests that the increase in adversarial data can provide a larger feature distribution in the data, thus allowing the network to learn the adversarial features. Moreover, this is also the insight of our study with more data, which is to showcase the generation of synthetic data. These findings collectively support the theory that adversarial patient positioning would contribute to segmentation model robustness. Furthermore, the addition of synthetic adversarial training data enhanced the consistency and performance of the deep learning-based bone segmentation model. Future works will include collection and testing on real CXRs which is shown to have the projection angle variation.

In radiomics, it is common to use image perturbations to determine features’ robustness and stability, with the goal of enhancing the reliability of radiomic analysis by using the robust features^45–48^. The perturbations includes rotation which also want to simulate the patient position variation during imaging. However, these studies mostly focus on CT images with rotation along z-axis. Our study focus on rotation along y-axis, as this patient rotation might happen during examination, and unlike z-axis rotation, it cannot be adjusted retrospectively. Also studies in deep learning models robustness have only been focusing on modifying the pixel intensity or translation, random cropping^10,16–18^, and have not bring forward to a clinical use case such as patient rotation. Therefore we propose the novel use of synthetic X-rays from CT for generating the adversarial patient positioned images for testing and training. Forward projected images could moreover reflect the actual attenuation effects of photon in X-ray beam on the anatomical changes when patient is rotated. Even though other generative view synthesis approaches might be applied, forward projection is a systematical reconstruction and a comparison of them is not our main focus. Extending beyond prior research^32–34^, our study further investigate in which aspect could the mixture of real and synthetic data-trained models outperform real data only trained models.

## Conclusion

This study has demonstrated the potential and effectiveness of applying adversarial synthetic X-rays generated from 3D photon-counting CT and TotalSegmentator annotations to quantify and increase robustness of bone segmentation models in X-ray. In real data-trained models, we found the models are less robust. As Dice scores increase in absolute value and spread with increasing internal patient rotation. Adding adversarial synthetic X-rays to training data reduces the variations and thus enhances model robustness. The focus of this study was on CXR and clavicle segmentation, but the underlying principle has great potential for applications in other domains including segmentation for hip replacement. Conclusively, we presented a systematic way of generating synthetic X-rays which can be used as an option to improve the robustness of deep learning models supplementary to standard approaches.

## Data Availability

The datasets generated during and/or analysed during the current study are not publicly available due to company data privacy, but might be available on reasonable request to the corresponding author.
